# The Impact on the Ecosystem Services Value of the Ecological Shelter Zone Reconstruction in the Upper Reaches Basin of the Yangtze River in China

**DOI:** 10.3390/ijerph15102273

**Published:** 2018-10-16

**Authors:** Zhilei Yu, Tianling Qin, Dengming Yan, Meijian Yang, Hexin Yu, Wanli Shi

**Affiliations:** 1 State Key Laboratory of Simulation and Regulation of Water Cycle in River Basin, China Institute of Water Resources and Hydropower Research (IWHR), Beijing 10038, China; yzl16@mails.tsinghua.edu.cn (Z.Y.); 18655058842@163.com (W.S.); 2 Department of Hydraulic Engineering, Tsinghua University (THU), Beijing 100084, China; 3 College of Environmental Science and Engineering, Donghua University, Shanghai 201620, China; 4 Department of Civil and Environmental Engineering, University of Connecticut, Storrs, CT 06269, USA; meijian.yang@uconn.edu; 5 JiLin Province Water Resources and Hydropower Consultaive Company of P R China, Changchun 130021, China; yuhexin1990@163.com

**Keywords:** ecological shelter zone reconstruction (*ESZR*), remote sensing, land use, the ecosystem service value (*ESV*), sustainable development

## Abstract

With regional socio-economic development and increasing population, the structure and function of terrestrial ecosystem environments on the earth’s surface have changed markedly. Ecological shelter zone reconstruction (*ESZR*) is an ecosystem restoration and conservation project, which aims to ensure the safety of the ecological environments of—regions and basins. We selected the upper reaches of the Yangtze River (from Yibin to Chongqing) as the study area and determined the connotation of *ESZR*. At the same time, the planning scope and construction content of the ecological barrier in this specific region have been preliminarily explored. Meanwhile, a set of related planning methods was proposed, the ecological effects of which were quantitatively assessed and confirmed through the calculation of *ESV*s. Compared with the conditions in 2005, the study showed that the value of the services of the whole ecosystem augmented significantly under the slope classification, increasing by 103.23%. At the same time, the land use pattern has been optimized, and the vegetation coverage has been enhanced. The *ESZR* can effectively improve the ecosystem service function of slope land (mainly slope > 25°) and the regional ecological environment, solve the rocky desertification of the study area and provide an effective decision in relation to supporting regional green sustainable development.

## 1. Introduction

With regional socio-economic development and increasing population, the structure and function of terrestrial ecosystem environments on the earth’s surface have changed markedly [1]. Environmental protection and construction could improve ecological environment conditions [2]. *ESZR* is an ecosystem restoration and conservation project, which aims to ensure the safety of the ecological environments of regions and basins [3,4,5]. It significantly ameliorates the ecological environment and improves the ecological service function [6,7].

**Figure 1:**
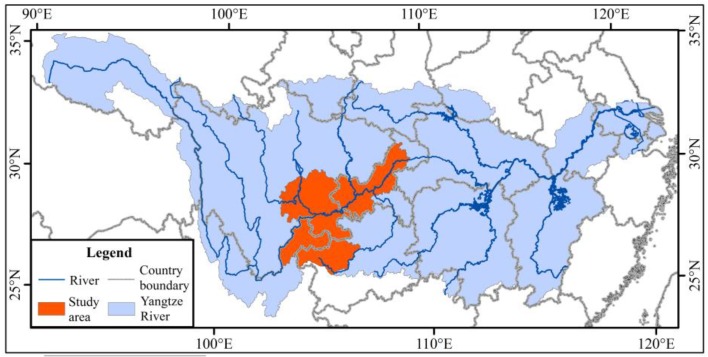
The location of the study area in the Yangtze River Basin.

“Ecological shelter” first emerged as a central term in 1999 [8]. The concept of an ecological shelter zone (*ESZ*) was discussed first in the upper reaches of the Yangtze River in 2001 [9]. The Upper Reaches Basin of the Yangtze River (from Yibin to Chongqing) covers the border areas of three provinces and one municipalities: Sichuan, Chongqing, Yunnan and Guizhou. It is the core area of the economic belt of the Upper Yangtze River and an important region of the West China Development Drive. The study revealed that the total gross domestic product (*GDP*) of Sichuan, Chongqing, Yunnan and Guizhou was less than 10% of the national total *GDP* in 2010 [10]. As a multi-city region, it has not comprehensively played an economic role in the whole Yangtze River economic belt (Figure 1). However, its ecological environment has experienced serious injury due to long-term excessive agricultural activities and urbanization [11,12].

The term “ecological shelter” has been used in China for several years, although it has always been vaguely or unclearly defined and lacks scientific elucidation. Some investigators affirmed that *ESZ* was a well-structured health ecosystem, which possessed self-sustaining and self-regulatory characteristics, with certain regional capacities [3,8,13,14,15]. It consists of different types of ecosystems, which provide protection for vulnerable parts of the environment. These parts of the environment usually offer certain ecological services, which have to be protected (e.g., the provisioning of drinking water or the productive function of soils). Outside of China, *ESZR* is equated with “ecosystem restoration” or “the restoration of protective ecosystem functions” [8]. Some researchers carried out qualitative research on its connotation, reasons, and measures, and proposed a division of functional districts, ecological assessment and ecological barrier construction, with different watershed scales [5,16,17,18,19]. However, agreement concerning the corresponding scientific questions, including the planning scope of the ecological protection barrier, the content and the value assessment of ecological construction, has not yet been achieved. Our study recommends the contents of *ESZR* and utilizes the ecosystem service value (*ESV*) to quantify the effects of *ESZR* with respect to the improvement of the ecological environment.

Concerning the *ESV*, Costanza et al. [20] identified the classification of global biosphere service functions and an assessment theory of *ESV*s. Meanwhile, Daliy et al. [21] proposed a relatively complete concept of *ESV*. *ESV*s are considered the natural conditions and effectiveness required for human survival, formed and maintained by ecosystems and the ecological process. Existing studies suggested that ecosystem service functions covered economic, social, cultural, ecological and productive functions [2,22]. Costanza et al. [20] grouped ecosystem services into 17 major categories. In China, several studies examined the economic values of ecosystem services, produced on various land use types, such as the forest ecosystem [23,24], the grassland ecosystem [25], and the aquatic ecosystem [26]. Besides, based on the rangeland biomass, Xie et al. [21] modified the unit area value of ecosystem services. Classifying land use types into two periods, Ran et al. [27] adjusted the unit *ESV* of diverse land use types. Given the human impact on ecosystems, we assessed the functional success of the *ESZR* by calculating the *ESV*.

In our study, we determined the connotation of *ESZR* based on Chinese characteristics. Meanwhile, the planning scope and specific construction contents of *ESZ* were preliminarily explored. The strategic position of the economic, social and ecological environment was studied on the basin scale by dividing the Yangtze River Basin radial zonation. Land adaptability was analyzed, and land use/vegetation coverage was adjusted. A set of planning methods of *ESZR* was proposed. By calculating the value of ecosystem services, the ecological effect of the *ESZR* was quantitatively assessed. Finally, the ecological service functions of slope land and vegetation coverage of forestland, grassland and orchards were effectively improved, and scientific references were provided to solve the rocky desertification of the study area and improve the regional ecological environment.

## 2. Study Area

We selected the upper reaches of the Yangtze River (from Yibin to Chongqing section) as the study area. It is located in the upper reaches of the Yangtze River Economic Belt, within 102°49′–109°15′ E and 26°15′–31°41′ N. It covers a total area of 164,000 km^2^, with 38.55% in Sichuan, 13.71% in Yunnan, 16.65% in Guizhou, and 31.09% in Chongqing. The development of the social economy in this region can not only promote the coordinated development of society and the economy of both the whole Yangtze River Basin and the region, but also promote the harmonious development of human and nature. The SFA [28] report shows that the upper reaches of the Yangtze River (from Yibin to Chongqing) have serious rocky desertification as well as many national poverty counties. The total rocky desertification is 22.3% in the upper reaches region of the Yangtze River, which includes Sichuan, Yunnan, Guizhou and Chongqing, as well as five state-level poverty-stricken counties and 37 special poor counties. Rocky desertification greatly impacts on ecological safety and economic growth in southwest China [29]. In total, 113,500 km^2^ had become rock deserts in Yunnan, Guizhou, Sichuan, Chongqing, Guangxi and Western Hunan by the end of 2000, and the economic loss exceeded tens of billions of yuan [30].

**Figure 2:**
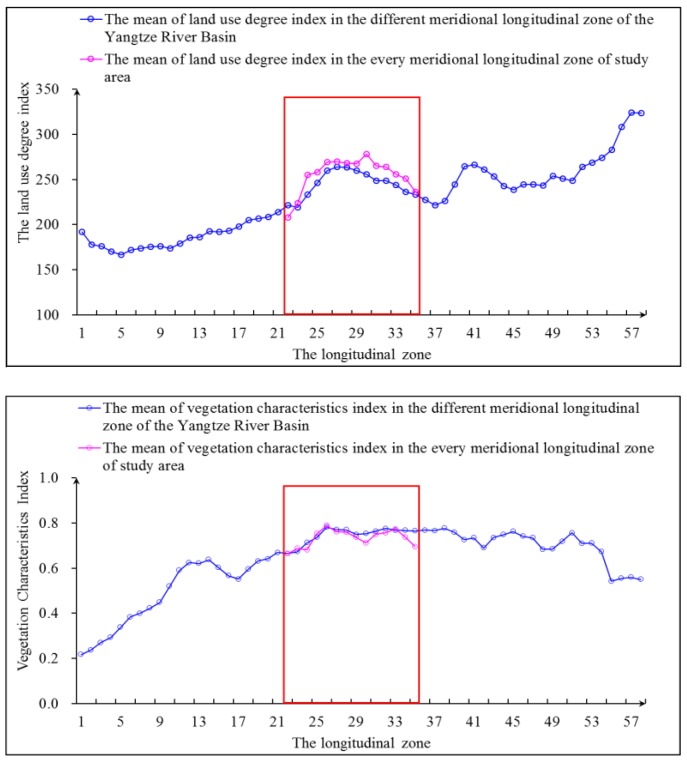
The land use degree index change and natural vegetation characteristics change in the study area and different meridional longitudinal zone of the Yangtze River Basin.

In addition, with respect to land use, the index degree of land use is relatively centered, and it has sufficient land use space for the economic development of the basin (Figure 2). With respect to vegetation, compared with the vegetation characteristics index in the same longitudinal zone of the Yangtze River Basin, the degree of natural vegetation coverage is relatively low in the research area, having a great promotion space for vegetation coverage (Figure 2). In general, land development and utilization changed more drastically in the study area than in the whole Yangtze River Basin. However, the degree of natural vegetation was relatively lower in the study area than in the whole Yangtze River Basin. Based on this economic and ecological situation, implementing *ESZR* and protecting vegetation of forests and grass are urgently needed to improve the rationality of land use and enhance the vegetation coverage.

**Figure 3:**
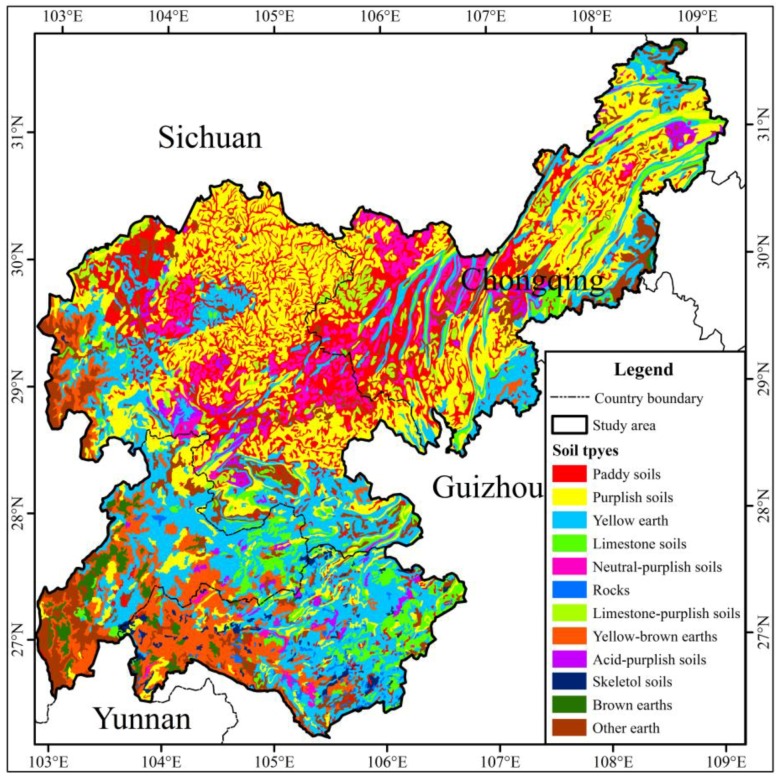
Map of soil types distribution.

**Figure 4:**
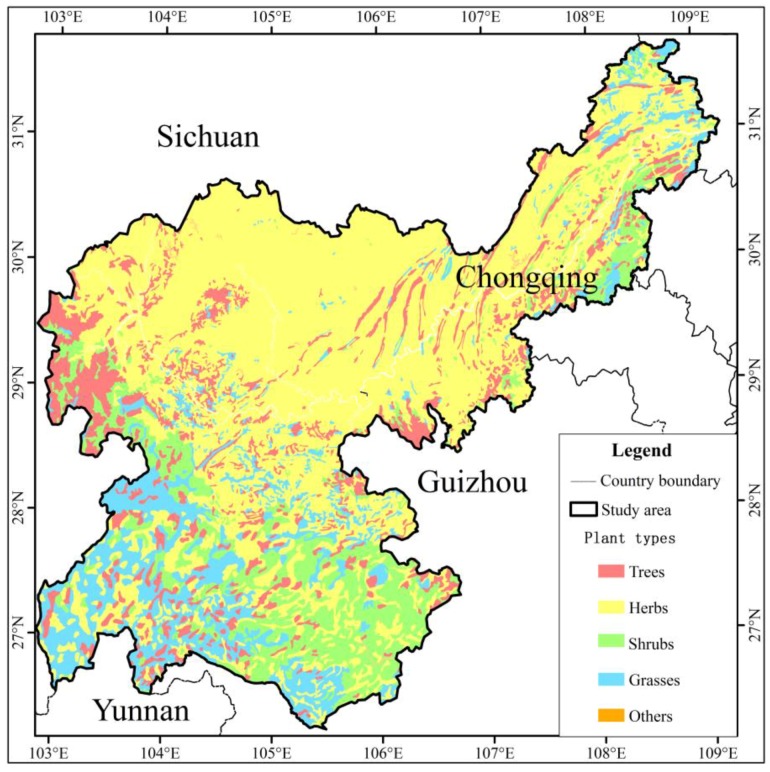
Map of main plant types distribution.

**Figure 5:**
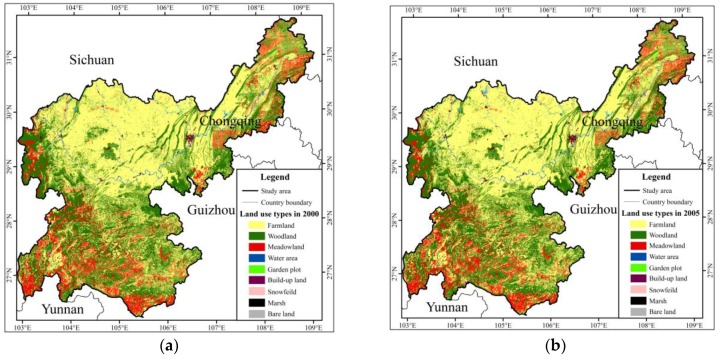
Land-use spatial distribution map: in 2000 (**a**); and in 2005 (**b**).

The research area has a moist, subtropical monsoon climate, which indicates apparent characteristics, such as hot summers, warm winters, high temperatures, long rainy seasons and little frost and snow. Soil types and distributions are complex in the study basin (Figure 3). The main plant types in the river basin are cultivated herbs, shrubs, brushes, coniferous and broadleaf tress, grasses and coniferous broad-leaved mixed trees (Figure 4). Considering the land use classification of *GB/T 21010-2007* [31], the ecological service function unit of the value calculation of ecosystem services and the regional situation of the study area, the land use types primarily include nine categories: cultivated land, orchard land, forest land, grassland, residential and urban construction land, waterbodies, marsh, snow and ice, and unused land (Figure 5).

## 3. Materials and Methods

Based on basic data, we assessed the overview of the study area, identified the construction scope of *ESZ* and the economic belt along the Yangtze River, evaluated the ecosystem services before and after *ESZR*, and optimized the layout of the land-use and vegetation coverage. The aim was to minimize the impact of human activities on the ecological environment.

### 3.1. Data

The main data gathered in this study include the national fundamental geographic information data, the Digital Elevation Model (*DEM*) at 1:250,000 and 1:50,000 scale, vegetation data at 1:100,000 scale, and soil and national land use data at 1:100,000 scale. The vegetation data are mainly composed of *MODIS* remote sensing data and the distribution of vegetation types, based on the national plant data. The soil and land use data were mainly extracted from the corresponding type distribution data. Land use in 2005 was considered the reference data.

### 3.2. Suitability Analysis

Human activities severely disturb the sustainability of land use and the ecosystem [32]. Suitability analysis could determine the fitness of land use. The Geographic Information System (*GIS*) and Analytical Hierarchy Process (*AHP*) are successfully utilized to assess the land suitability [33,34,35,36]. The *GIS*-based *AHP* evaluates the suitability analysis needed to consider numerous sophisticated and critical criteria. Our study considered topographic, soil and meteorological criteria that affect the suitability of land use, including the cropland, orchard, forestland, grassland and built-up land. The specific parameters cover the elevation, slope, surface roughness (microrelief), soil types, average annual precipitation and mean annual temperature. According to the *AHP* method, we weighted these elements using ArcGIS 10.2 (Environmental Systems Research Institute, Inc., Redlands, CA, USA). Then, we derived the total score for each land use unit by integrating the criteria score and criteria weights [34]. The Weighted Linear Combination (*WLC*) was applied to calculate the composite weights using the following expression:

(1) $$Gd=\sum_{i=1}^{n}V_{ij}\times W_{i}\;(j=1,2,3,\ldots ,m\;i=1,2,3,\ldots ,n)$$

where *G_d_* presents the total score of the *j*th land use unit; *V_ij_* is the score for the *i* criterion in the *j*th land unit; *W_i_* is the *i*th criterion weight that is determined by the *AHP* method; *n* is the number of the criteria; and *m* is the number of land units in the study area.

According to the spatial distribution of the total score, the suitability categories are divided into five different classes [37,38,39]: (A) most suitable; (B) highly suitable; (C) moderately suitable; (D) marginally suitable; and (E) least suitable.

Finally, the suitability of various land use types was derived for the study area, including cultivated land, forest land, orchard land, grassland and residential and urban construction land.

### 3.3. Evaluation of the NDVI and F_c_

Based on *MODIS* remote sensing, the Normalized Difference Vegetation Index (*NDVI*) was extracted using the *ENVI4.7* platform, and the Fractional Vegetation Cover (*F_c_*) and rating were divided [40,41]. The status of the vegetation coverage in the study area was analyzed and evaluated using *NDVI* and *F_c_* [42,43].

*NDVI* is a remote sensing indicator that reflects a state of land vegetation growth, limited to the range of [−1, 1]. *NDVI* is greater than zero in places covered with vegetation. *NDVI* increases with the increase of vegetation cover. The calculation formula is as follows:

(2) $$NDVI=\frac{NIR-VIS}{NIR+VIS}$$

where *NIR* presents the reflectance of the remote sensing channel near the infrared bands; and *VIS* is the reflectance of the remote sensing channel near the visible light wave band.

*F_c_* refers to the ratio of the vertical projection area of the vegetation canopy to the total soil area, namely, the ratio of plant to land use. The calculation formula is as follows:

(3) $$F_{c}=\frac{NDVI-NDVI_{\min}}{NDVI_{\max}-NDVI_{\min}}$$

where *NDVI*_max_ presents the maximum value of *NDVI* in the study area; and *NDVI*_min_ is the minimum value of *NDVI*. According to the results derived by Zhou et al. [40], when the *F_c_* ratio is greater than 60%, the vegetation coverage class is defined as the high class (A); 30% ≤ *F_c_* ≤ 60% means the suitably class (B); 15% ≤ *F_c_* < 30% presents the moderate class (C); 5% ≤ *F_c_* < 15% expresses the marginal class (D); and an *F_c_* ratio less than 5% means a weak coverage (E).

### 3.4. Derivation of the Scope of the ESZR

To solve rocky desertification and improve the regional ecological environment, we derived the *ESZR*. We adjusted the land use/vegetation cover pattern by using engineering and non-engineering measures to arrange and optimize the ecosystem structure to improve *F_c_* and *ESV*. The derivation method of the *ESZR* is as follows.

We operated the *DEM* to generate a map of the slope, surface roughness, and digital river network in ArcGIS 10.2. We generated the buffer layers of the main stream, secondary tributary and road. Then, we carried out an overlay analysis to obtain the alternative area of the economic belt by inputting all layers and adding them together. Furthermore, we considered the region (slope < 15° and surface roughness < 0.5) without cropland as the economic industrial concentration area. Finally, based on the concentration region, we derived the economic belt along the Yangtze River.

**Figure 6:**
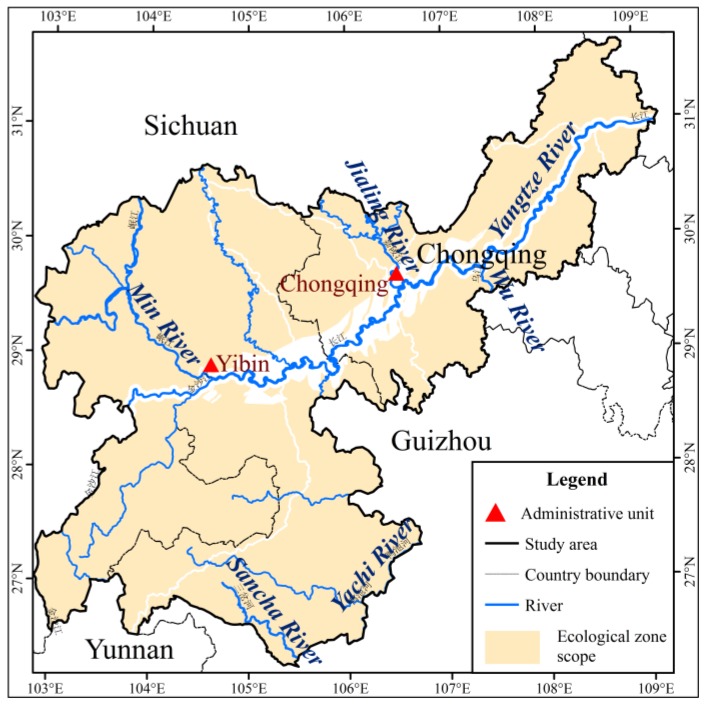
The scope of *ESZR*.

In consideration of the results of the land use suitability evaluation, and referring to some related literature materials [44,45], the study area was divided into two layers. One has a higher slope (>25°) and the slope of the other layer is <25°. The vegetation coverage was vectorized and overlaid with land use data to obtain the land use and the level of the vegetation cover. Then, removing the parts of the buffer and economic zone along the Yangtze River, the planning range of the ecological barrier was finally generated (Figure 6).

### 3.5. ESZR Scheme

**Figure 7:**
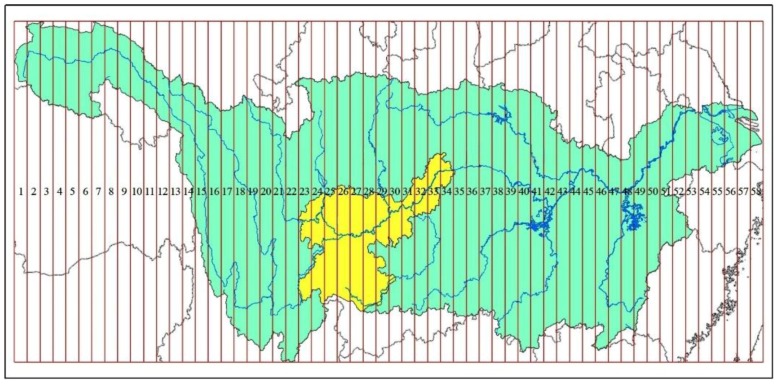
Radial zoning map of the study area and Yangtze River Basin (the green part is the Yangtze River Basin, and the yellow part is the study area.).

The Yangtze River Basin was divided into a 58 meridional zones (the interval was 50 km), from left to right. The study area was located in the No. 22–No. 35 band. It was the transition zone, from the second step to the third step of China’s terrain. It is a multi-city region located in the upper reaches of the Yangtze River economic belt. It is the crucial region of the central and western development in China. It has an extremely important socio-economic and ecological environment status in the entire Yangtze River Basin (Figure 7). Combined with the regional natural vegetation, the *ESZ* for the lower reaches of the Yangtze River was provided by adjusting the land use pattern and improving the distribution rate of the artificial forest and natural vegetation.

**Figure 8:**
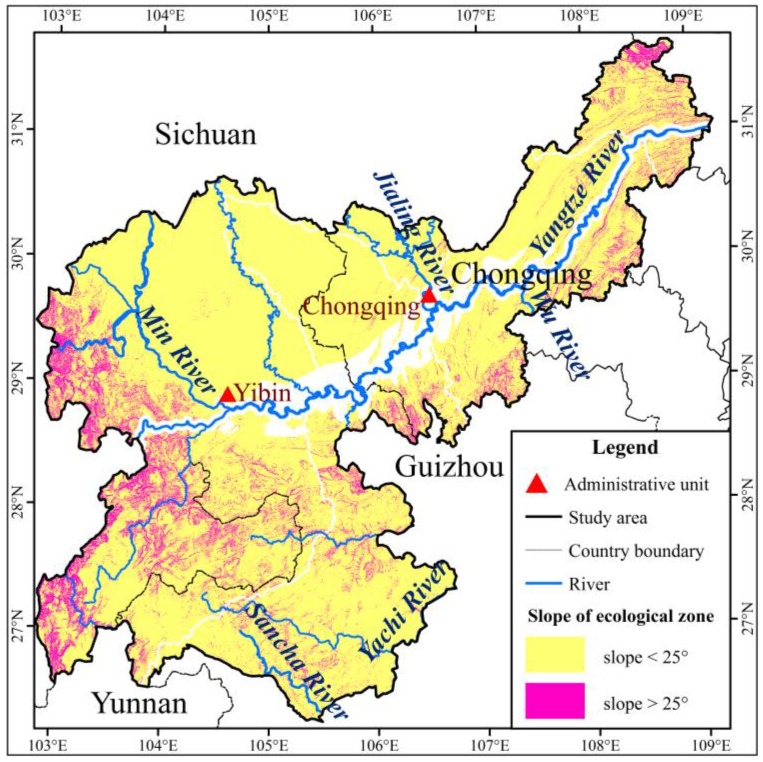
Overall pattern of ecological protecting-barrier construction planning.

The planning scope of the *ESZR* was predominantly in regions with a slope > 25°, and some areas had slopes < 25° (Figure 8). Croplands (slope > 25°) must be changed into ecological forestland or meadows. Engineering measures were given priority in the conversion of cropland into forestland or grassland. Meanwhile, the integral *F_c_* needs to be improved.

The different planning methods of *ESZR* are shown in Table 1. To adjust and improve the structure of slope land, vegetation coverage ratio, and land use in the study area, we considered seven scenarios. Among them, the construction of a slope classification was achieved through the following steps: (1) conversion of the cropland (slope > 25°), grassland (slope > 25°), and built-up land (without road, slope > 25°) to orchards; (2) conversion of all unused land (slope > 25°) to forestland with a high vegetation coverage; (3) conversion of all unused land (slope < 25°) to orchards; and (4) enhancement of the whole *F_c_* ratio of forestland above 60%, or at least above 30%.

### 3.6. Evaluation of Ecosystem Services Values

Ecosystem services have multiple functions, which sustain and fulfill human survival, including provisional, regulatory, cultural and support services [20,46,47,48]. These services have been greatly altered by land use and land cover changes [49,50]. Our study quantified and analyzed the variation of ecosystem services, before and after the *ESZR*, by calculating the corresponding *ESV*s. Referring to the *ESV* assessment results [20,25,27], we calculated the total *ESV* in the study area as follows:

(4) $$ESV=\overset{8}{\underset{i=1}{\sum }}\overset{17}{\underset{j=1}{\sum }}P_{ij}\times A_{i}$$

where *ESV* is the estimated total ecosystem services value; *P_ij_* is the adjusted *ESV* per unit area for land use type (10^8^ yuan·(hm^2^·a)^−1^); and *A_i_* is the area for the land use category *i* (hm^2^). Based on the specific situation in China, Ran et al. [25] adjusted the *ESV* per unit area of different land use categories (Table 1). The ecosystem services and functions listed in Table 2 refer to the categories derived by Costanza et al. [20].

## 4. Results

### 4.1. Land Use Suitability Characteristics

Compared to the situation of land use in 2000, the overall land use changed slightly in 2005. The area of cropland, orchards, forestland, waterbodies, marshes and snow area increased by 0.07%, 16.10%, 1.08%, 0.91% and 41%, respectively, while grassland, built-up land, and unused land decreased by 3.57%, 3.57% and 3.57%, respectively, in 2005 (Table 3).

**Figure 9:**
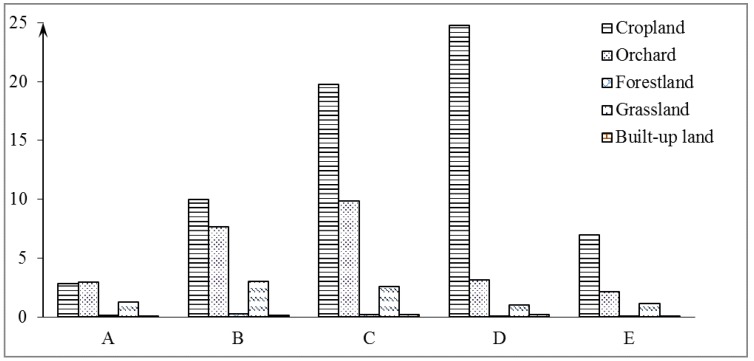
The proportion of the land use suitability area of different land use patterns (the proportion of the land use suitability grade is divided into A–E in Figure 8: (A) most suitable; (B) highly suitable; (C) moderately suitable; (D) marginally suitable; and (E) least suitable).

**Figure 10:**
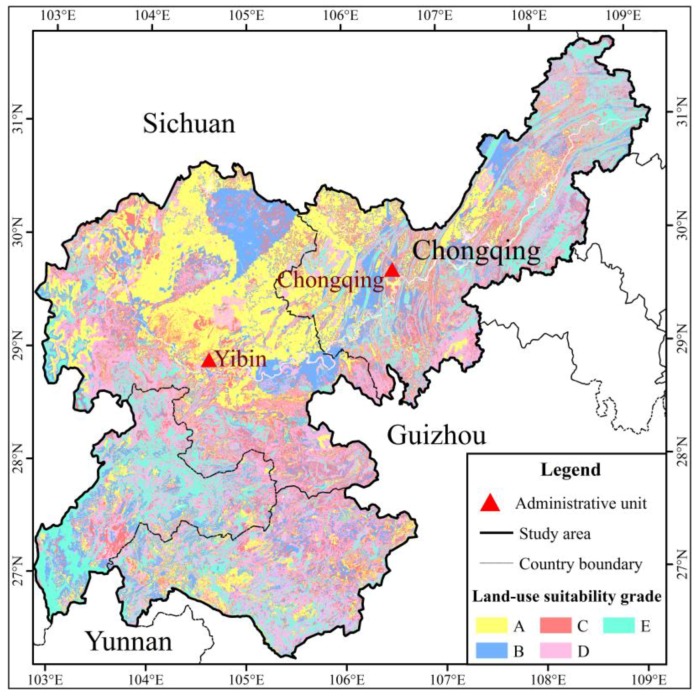
Distribution of land-use suitability grade.

The suitability evaluation results in relation to land use in 2005, except for watershed and unused land, are presented in Figure 9 and Figure 10. About 1.17% of the land use area had the highest suitability (A), 3.40% of the land use area had high suitability (B), 5.28% of the land use area had moderate suitability (C), 4.72% of the land use area had marginal suitability (D), and 1.66% of the land use area had the lowest suitability (E) (Figure 7). The entire land use area was considered unsuitable for cropland, including areas of D and E, most of which was in the southwest and northeast parts of the study area (Figure 8). Moreover, the slopes in these regions were >25°. The areas of A, B and C were concentrated the northern part of the study area. The suitable areas of A and B were relatively little, accounting for about 30% of the total study area. The whole land use suitability was not high, and the rationality of land use was low.

### 4.2. Vegetation Coverage Characteristics

**Figure 11:**
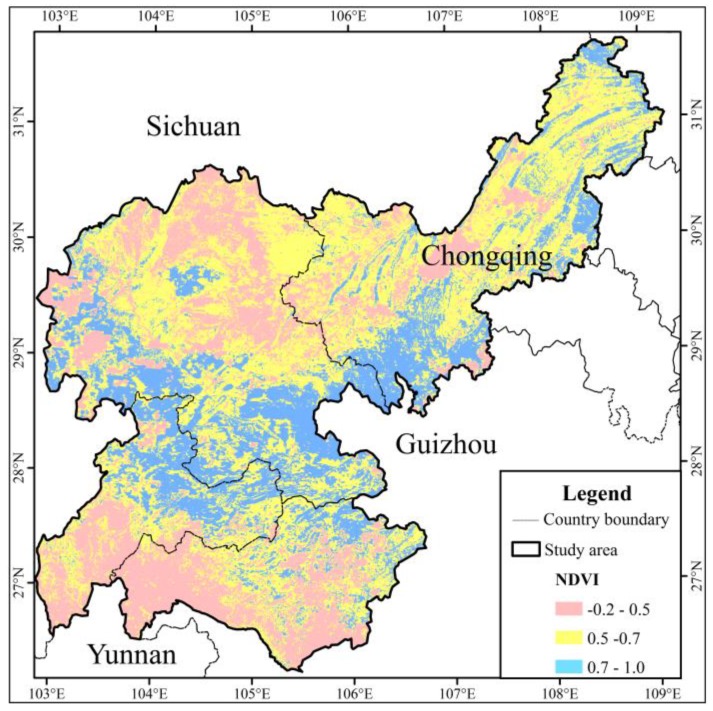
Map of normalized difference vegetation index.

**Figure 12:**
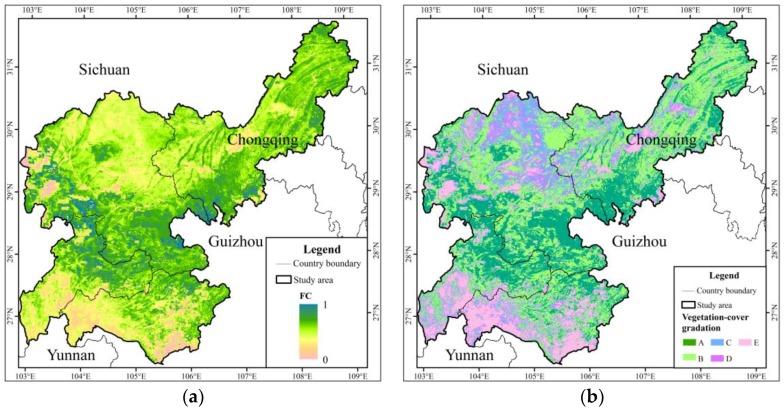
Fractional vegetation cover (**a**); and gradation distribution (**b**) ((A) first class; (B) second class; (C) third class; (D) fourth class; and (E) fifth class).

The average *NDVI* was 0.585 in the study area. This indicated that the overall regional state of vegetation growth was at the medium level, and the vegetation coverage should be improved. From the perspective of spatial distribution characteristics, the *NDVI* was low in the southeastern part of the Sichuan, northeast part of Yunnan, and southwest part of Guizhou, while it was high in the southern part of Sichuan, south and northeast of Chongqing City, and northwest of Guizhou (Figure 11). As shown in Figure 12, the district of the first vegetation coverage class was relatively concentrated in the central part of the study area. The second class was mainly distributed in the central and northeast parts of the study area. The southern part of the Sichuan was dominated by the third and fourth classes of vegetation-cover (i.e., the northern part of the study area), while the fifth class of vegetation coverage was given priority in the northeastern part of Yunnan and the western part of Guizhou.

### 4.3. Evaluation of the ESVs

(1) Current Services Value of the Area

**Figure 13:**
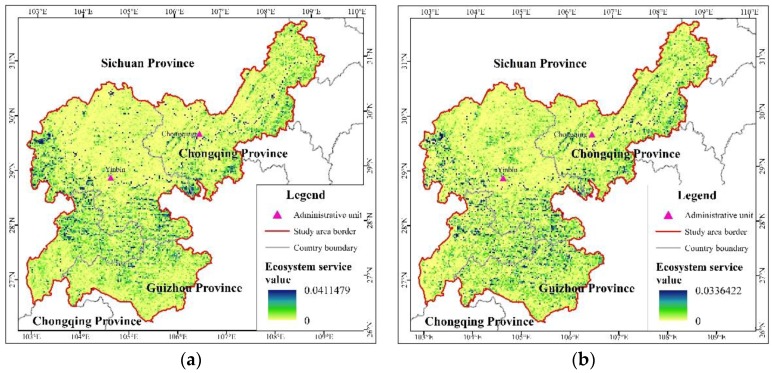
Spatial distribution of *ESV*: before *ESZR* (**a**); and after *ESZR* (**b**).

Based on the land use and vegetation cover distribution, the total ecological services value was about 42.662 billion yuan in the upper reaches of the Yangtze River in 2005 (Figure 13a). Forestland had the highest value, accounting for about 49.4%. Due to the small area and low unit value, the ecological value proportion of snow was nearly zero (Table 4). There was a large difference in the unit *ESV* of each land use type (Table 2). In contrast, the overall ecosystem services value ranks of each land use type were as follows, from high to low: wetlands, waterbodies, forestland, orchards, grassland, arable land and snow. The area of cultivated land was the largest due to its low *ESV*, thus its gross ecosystem services value was lower. The value of waterbodies was small, although their gross value was higher because of their higher unit value of ecological services. Generally, the *ESV* mainly depended on the distribution area of forest and water.

(2) Comparison of the Regional Ecosystem Services Value of Different Planning Programs

With a view to elucidating different ecological planning and construction programs, the corresponding regional *ESV*s were obtained. Their growth values are shown in Table 5. It can be seen that the *ESV* improved most significantly under the slope classification construction of *ESZR* (Figure 13b). Moreover, this *ESZR* had an outstanding effect in improving the ecosystem service function of cropland, especially in the slope region (>25°).

### 4.4. Land Use Optimization under Ecological Barrier Construction

From the perspective of the whole and the part, land use pattern could be compared and analyzed, before and after *ESZR*. By contrast, the optimization effect of land use pattern was obvious, and the rationality of land use was improved after *ESZ*. This indicated that there was a certain feasibility of putting the slope classification construction scheme into practice. The main results were as follows:

(1) The Overall Pattern Optimization

Compared with the situation of the land use pattern in 2005, overall, cropland, grassland, built-up land, waterbodies and unused land showed a decreasing trend, reducing by 1.78%, 1.8%, 0.01%, 0.38% and 0.03%, respectively, with the recommended plan. At the same time, orchards increased by 3.6%, and wetlands added 0.39% (Table 6).

(2) Slope Gradient Greater than 25 Degrees

Compared with the situation of the land use pattern in 2005, the proportion of cropland, grassland, built-up land and unused land decreased by 19.16%, 19.37%, 0.03% and 0.02%, respectively, in the region with slopes > 25°, while orchards rose by 38.57% (Table 7).

(3) Slope Gradient Lower than 25 Degrees

Compared with the present situation of the land use pattern, the proportion of waterbodies and bare land dropped by 0.48% and 0.03%, respectively, in this area. Orchards and wetlands were enhanced by 0.03% and 0.47%, respectively, in this region (Table 8).

### 4.5. Fractional Vegetation Optimization under Ecological Barrier Construction

**Figure 14:**
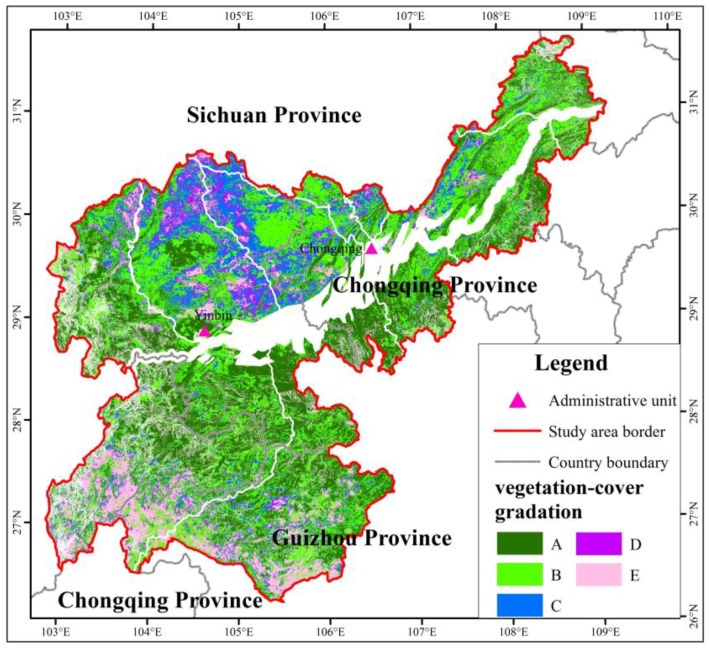
*F_c_* Classification of ecological barrier construction under recommended program.

Compared with the *F_c_* ratio in 2005, the proportion of A class vegetation coverage increased by 9.34% under slope classification construction. The proportion of B, C, D and E classes of vegetation coverage was reduced by 1.68%, 2.64%, 1.25% and 3.77%, respectively (Table 9 and Figure 14). To some extent, the whole vegetation coverage of the study area was improved by the reconstruction of *ESZ*, and the ecological environment was also enhanced.

## 5. Discussion

*AHP* is one of most comprehensive techniques for analyzing land suitability [35,39]. Parry et al. [32] analyzed six elements to obtain three categories of urban suitability. Aburas et al. [35] used 17 factors, including main and sun factors, to derive five suitability categories. Zabihi et al. [51] utilized 14 factors to obtain the suitable regions for future citrus planning. We also selected six geographic factors to assess the land-use suitability scope of the ecological belt zone. Demesouka et al. [52] pointed out that the criteria parameters were strongly related to the study area’s features. In our study, we only considered the natural geographic elements because ecological belt zone construction focused on natural ecological restoration. Consistent with study of Yu et al. [34], unsuitable lands were dominated by steep slopes (slope > 25°). Thus, we suggested mainly converting these regions (slope > 25°) to *ESZR*.

The *ESZR* aims at solving rocky desertification and improving vegetation cover to ameliorate the regional ecological environment in the study area. The study demonstrated that rocky desertification was concentrated in Yunnan, Guizhou, Chongqing, Sichuan, Guangdong, Hunan, Hubei and Guangxi in southwest China [30], which was consistent with the rocky desertification distribution in our study. Meanwhile, vegetation cover is a crucial part of terrestrial ecosystems, and vegetation indices are well-known and widely used to qualitatively and quantitatively evaluate vegetation covers [53]. Combining vegetation covers with land use in our study, *ESZR* methods proposed the enhancement of the regional vegetation coverage ratio, optimization of the structure of land utilization and protection of the ecological environment, which was close to the purposes of Feng et al. [2].

Terrestrial ecosystems are complicated, and different types of ecosystems (e.g., forestland and cropland) interact [54,55,56]. For the *ESV*s, forestland, grassland and waterbodies played significant roles in ecological restoration and the protection of the environment [10,57]. The *ESV* had a continuously declining tendency from 1990 to 2010 in China (Li and Wang, 2016). The *ESV* was obviously improved after the *ESZR* in our study. Thus, reconstructing the *ESZ* in the Upper Reaches Basin of the Yangtze River is of profound significance. Furthermore, for rationality and universality, our results could be applied to other watersheds to determine whether it is useful for the improvement of the ecological environment in our further work.

## 6. Conclusions

(1) The whole land utilization ratio was low, and the rationality of land use was not high. The most highly suitable area was relatively low. This accounts for about 30% of the total study area, and other land use types accounted for nearly 70%. Cropland with slopes > 25° was a marginal region and the least suitable. The overall regional state of vegetation growth was at a medium level, and the vegetation coverage should be improved.

(2) The planning scope of the *ESZR* principally covered regions with a slope gradient > 25°, and some areas had slopes < 25°. There were different schemes concerning the *ESZR*. Among them, the regional ecological value had increased by 103.23%, and its total value reached 86.703 billion yuan under the slope classification construction. This was mainly caused by the reduction in the area of cropland, built-up land and orchards; the conversation of all unused land to forestland and orchards; and the enhancement of the forestland area on an original vegetation coverage basis.

(3) The area of orchards and wetlands increased as the slope classification was constructed. Meanwhile, the cropland, grassland, built-up land, waterbodies and unused land decreased. By contrast, the optimization effect of the land use pattern was obvious, and the rationality of land use was improved. The service function of land in slope fields was enhanced, and the whole vegetation coverage was improved as well.

(4) The proportion of A class vegetation coverage increased by about 10% under the slope classification construction. Others were reduced to different extents. The whole vegetation coverage of the study area was improved, and the ecological environment was enhanced.

## Figures and Tables

**Table 1:** The planning of the *ESZR*.

Planning	Groups	Slope Gradient	Description	
Converting cropland to forestland	i	>25°	The proportion of cropland converted was 30%.	
	ii	>25°	The proportion of cropland converted was 50%.	
	iii	>25°	The proportion of cropland converted was 80%.	
Converting cropland to grassland	iv	>25°	The proportion of cropland converted was 30%.	
	v	>25°	The proportion of cropland converted was 50%.	
	vi	>25°	The proportion of cropland converted was 80%.	
Slope classification construction	vii	>25°	Converting the cropland, grassland, and built-up land (without roads) to orchards	The whole *F_c_* ratio of forestland should be enhanced above 60%, or at least above 30%, which means converting the forestland vegetation coverage classes C, D and E to B or A, and the vegetation coverage classes A and B remain unchanged.
			Converting all unused land to forestland with a high vegetation coverage	
		<25°	Converting all unused land to orchards	

**Table 2:** *ESV* per unit area of different land use types (unit: yuan·(hm^2^·a)^−1^).

Land Use	Cropland	Orchard	Forestland	Grassland	Built-Up Land	Waterbodies	Wetlands	Snow
Gas regulation	12.39	61.24	82.6	39.87	—	8.26	2188.9	16.52
Climate regulation	—	363.85	726.88	0.83	—	8.26	82.6	16.52
Disturbance regulation	41.3	344.03	413	275.06	—	—	59,802.4	—
Water regulation	—	19.64	24.78	14.51	—	44,975.7	247.8	8.26
Water supply	—	115.15	—	230.3	—	17,486.42	17,486.42	8.26
Erosion control and sediment retention	—	86.62	41.3	131.94	—	—	82.6	—
Soil formation	4.13	43.52	82.6	4.44	—	—	—	—
Nutrient cycling	4.13	8.67	16.52	0.83	—	8.26	8.26	—
Waste treatment	—	683.7	718.62	648.77	—	5492.9	13,703.34	—
Pollination	115.64	139.46	165.2	113.71	—	—	—	—
Biological control	198.24	68.79	33.04	104.55	—	—	—	—
Refugia	—	91.82	165.2	18.45	—	—	330.4	—
Food production	446.04	366.61	413	320.22	—	338.66	247.8	—
Raw production	4.13	106.48	206.5	6.47	—	—	247.8	—
Genetic resources	4.13	7.85	14.87	0.83	—	—	16.52	—
Recreation	—	170.61	297.36	43.87	82.6	1899.8	338.66	8.26
Cultural	—	34.97	16.52	53.43	82.6	—	165.2	—

**Notes:** (1) “—” denotes lack of data or absence of such ecological services because of the low ecological value in traffic land and bare land (including saline-alkali soil and sand). The value of ecosystem services has not been taken into consideration. (2) Built-up land refers to the residential and urban construction land.

**Table 3:** Area of each land use type in the study area in 2000 and 2005 (unit: %).

Land-Use Types	Cropland	Orchard	Forestland	Grassland	Built-Up Land	Waterbodies	Unused Land	Wetlands	Snow
In 2000	53.35	0.45	32.40	11.87	0.79	1.11	0.04	0.00	0.00
In 2005	53.39	0.52	32.75	11.45	0.75	1.12	0.03	0.00	0.00

**Table 4:** The total and proportion of *ESV* of different land use types in the region (unit: 10^8^ yuan).

Land Use	Cropland	Orchards	Forestland	Grassland	Built-Up Land	Waterbodies	Unused Land	Wetlands
Total value	72.06	2.31	186.82	37.75	0.20	128.35	0.22	0.00
Proportion (%)	17.07	0.53	43.11	8.85	0.05	30.35	0.05	0.00

**Table 5:** Comparison of the *ESV*, before and after *ESZR*.

Planning	Groups	Slope Gradient	The Proportion Converted	*ESV* (10^8^ yuan)	
				After the *ESZR*	*ESV* Improved
Converting cropland to forestland	i	>25°	30%	496.17	3.17
	ii	>25°	50%	498.3	5.3
	iii	>25°	80%	501.87	8.47
Converting cropland to grassland	iv	>25°	30%	493.91	0.91
	v	>25°	50%	494.6	1.6
	vi	>25°	80%	495.63	2.63
Slope classification construction	vii	—	—	867.03	440.41

**Table 6:** Comparison of land use types between the present situation and alternate solutions (unit: %).

Land Use	Cropland	Orchards	Forestland	Grassland	Built-Up Land	Waterbodies	Unused Land	Wetlands	Snow
Current situation	53.39	0.52	32.75	11.45	0.75	1.12	0.03	0.00	0.00
Recommendation	51.61	4.12	32.75	9.65	0.74	0.74	0.00	0.39	0.00
Comparison	−1.78	3.6	0	−1.8	−0.01	−0.38	−0.03	0.39	0.00

**Table 7:** Area ratio comparison of land use types between the present situation and region with slopes > 25° under the recommended program unit: %).

Land Use	Cropland	Orchards	Forestland	Grassland	Built-Up Land	Waterbodies	Unused Land	Wetlands	Snow
Current situation	19.16	0.21	61.10	19.37	0.03	0.10	0.02	0.00	0.00
Recommendation	0.00	38.78	61.12	0.00	0.00	0.10	0.00	0.00	0.00
Comparison	−19.16	38.57	0.02	−19.37	−0.03	0.00	−0.02	0.00	0.00

**Table 8:** Area ratio comparison of land use types between the present situation and region with slopes < 25° under the recommended program (unit: %).

Land Use	Cropland	Orchards	Forestland	Grassland	Built-Up Land	Waterbodies	Unused Land	Wetlands	Snow
Current situation	54.81	0.46	32.06	11.46	0.54	0.65	0.03	0.00	0.00
Recommendation	54.81	0.49	32.06	11.46	0.54	0.17	0.00	0.48	0.00
Comparison	0.00	0.03	0.00	0.00	0.00	−0.48	−0.03	0.47	0.00

**Table 9:** Comparison of the *F_c_* classification between the recommended program and status quo (unit: %).

*F_c_* Classification	A	B	C	D	E
Current situation	26.79	38.00	14.96	6.34	13.92
Recommendation	36.13	36.32	12.32	5.09	10.15
Comparison	9.34	−1.68	−2.64	−1.25	−3.77
